# Management of Refractory Gastrointestinal Bleeding in Hereditary Hemorrhagic Telangiectasia with Bevacizumab

**DOI:** 10.1155/2021/2242178

**Published:** 2021-06-29

**Authors:** Muaaz Masood, Michael Coles, Humberto Sifuentes

**Affiliations:** ^1^Department of Internal Medicine, Medical College of Georgia at Augusta University, Augusta, Georgia, USA; ^2^Department of Gastroenterology and Hepatology, Medical College of Georgia at Augusta University, Augusta, Georgia, USA

## Abstract

Hereditary hemorrhagic telangiectasia (HHT) is an autosomal dominant disorder resulting in vascular malformations of several organs including the pulmonary, cerebral, and gastrointestinal systems. One sequela is recurrent gastrointestinal (GI) bleeding. Bevacizumab (Bev) is emerging as an effective treatment of recurrent gastrointestinal bleeding in HHT. Bev is a recombinant monoclonal antibody that inhibits vascular endothelial growth factor (VEGF), an integral part of angiogenesis.

## 1. Introduction

Hereditary hemorrhagic telangiectasia (HHT), also known as Osler–Weber–Rendu syndrome, is an autosomal dominant disorder which is characterized by the abnormal formation of blood vessels [1]. HHT affects approximately 1 in 5000 individuals in North America [2]. Its prevalence has been underreported due to variable penetrance [3].

Several genetic mutations have been identified in HHT [4]. The most common mutations are in *ENG*, *ACVRL1*, and *SMAD4* which encode proteins that are part of the transforming growth factor beta (TGF-*β*) pathway in vascular endothelial cells [2]. Studies have demonstrated that patients with HHT have abnormal levels of TGF-*β* and vascular endothelial growth factor (VEGF) which are key players in angiogenesis [3, 5]. Mutations in these pathways result in abnormal vessel development and arteriovenous malformations (AVMs) [6].

AVMs can be present in mucocutaneous membranes (i.e., lips, tongue, buccal mucosa, or fingers) as well as in the lungs, brain, and GI tract [5]. Bleeding of AVMs is common and typically presents as epistaxis or symptoms related to iron deficiency anemia [3]. Recurrent GI bleeding is present in up to one-third of patients [7]. These patients often fail to respond to conventional treatments [8].

Bevacizumab (Bev) is a recombinant monoclonal antibody that inhibits VEGF [9]. Bev is emerging as an efficacious treatment for recurrent GI bleeding in patients with HHT [5, 9–14]. We present a case of a 67-year-old Caucasian female with a history of HHT, GI AVMs, and multiple admissions for recurrent GI bleeding who failed conventional therapy and had an excellent clinical response to Bev infusions.

## 2. Case Presentation

**Figure 1:**
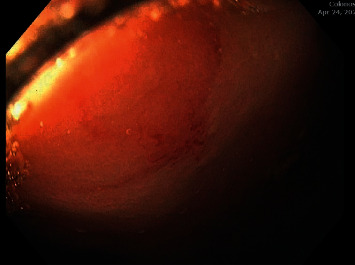
Arteriovenous malformation in the cecum.

**Figure 2:**
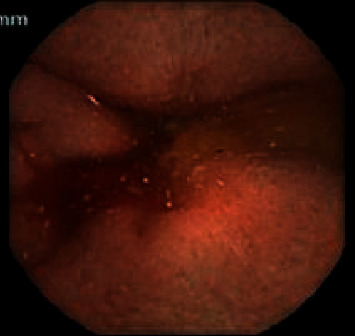
Intraluminal bleeding in the duodenum on video capsule endoscopy.

**Figure 3:**
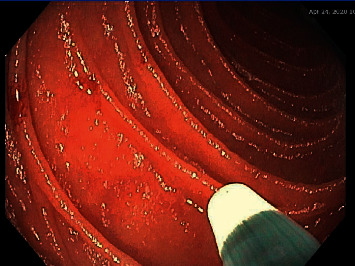
Two arteriovenous malformations in the jejunum.

A 67-year-old Caucasian female with a history of HHT with GI AVMs and no pulmonary or hepatic AVMs required multiple hospital admissions for recurrent gastrointestinal bleeding. In one calendar year, the patient had 8 admissions for gastrointestinal bleeding and required a total of 14 units of packed red blood cells (pRBC) transfused. Endoscopic evaluation showed recurrent gastric, duodenal, jejunal, and colonic AVMs (Figures 1–3) which were treated with argon plasma coagulation. The patient was tried on tranexamic acid and aminocaproic acid without improvement. She was started on Bev 5 mg/kg every 2 weeks for a total of 6 infusions over 3 months. She had marked improvement in symptoms and a decrease in the frequency of bloody stools since induction. With regard to anemia, her hemoglobin level recovered to 13 g/dL from 9 g/dL after receiving the 6 Bev infusions. Her hemoglobin level remained at its peak of 13 g/dL for approximately 2 months. She had just one hospitalization for GI bleeding and required only one pRBC transfusion since induction with Bev. The plan is for Bev to be administered if the patient's hemoglobin decreased 1.5 g/dL from its peak. It was determined that the patient would likely require a dose of Bev once every 2 months with serial monitoring of her hemoglobin levels given the severity of her disease.

## 3. Discussion

HHT is a multisystemic disease which results from mutations in genes that regulate angiogenesis [5]. AVMs are a hallmark of the disease and can occur in the gastrointestinal, cerebral, pulmonary, and integumentary systems [1]. The most common symptoms include epistaxis, symptomatic anemia, GI bleeding, and mucocutaneous telangiectasias [3]. The diagnosis is clinical with three or more of the following features which must be present to establish the diagnosis: epistaxis, mucocutaneous telangiectasias, visceral AVMs, and a family history in a first-degree relative [2]. Genetic testing (i.e., SMAD4, ACVRL1, ENG, and GDF2) is not only performed for confirmation in individuals in whom there is a high suspicion for the disease but also may define the risk of various types of AVMs [4, 15]. A study by Canzonieri et al. explored the prevalence and distribution of telangiectasias in relation to their genotype. Patients with mutations in ENG (HHT-1), ACVRL1 (HHT-2), and SMAD4 (in association with juvenile polyposis syndrome) underwent endoscopic evaluation, and it was discovered that patients with ENG mutation (HHT-1) had a higher rate of multiple telangiectasias with a higher prevalence of telangiectasias in the duodenum [15]. Genetic testing in our patient was negative for ALK1, ENG, SMAD4, RASA1, and GDF2. However, our patient reported a childhood history of epistaxis. She also had a family history of GI bleeding and epistaxis in her father. Examination of the patient was notable for mucocutaneous telangiectasias on her lips and thumb. Endoscopic evaluation revealed recurrent GI AVMs.

AVMs in the GI tract typically occur in the stomach and duodenum and most commonly present as GI bleeding after 40 years of age [7]. AVMs in the liver are typically between hepatic arteries and portal veins and may manifest as symptoms of pulmonary or portal hypertension, heart failure, abdominal pain, or encephalopathy [16]. There may also be abnormalities in cardiac biomarkers or liver function tests [17]. HAVMs are more prevalent in patients with mutations in ACVRL1 (HHT-2) [18]. The current guidelines recommend screening for HAVMs with diagnostic imaging in patients who are symptomatic and in patients whom HHT is suspected or definite [17].

Recurrent GI bleeding in HHT is debilitating and is associated with a decreased quality of life [1]. Chronic bleeding episodes also result in increased healthcare costs, repeated hospital admissions, endoscopic interventions, and pRBC transfusions [8]. Repeated endoscopic ablation of GI AVMs is not recommended due to poor long-term results and complications [19].

Bev has established efficacy in the treatment of inflammatory disorders and in various cancers in which the mechanism of action is inhibition of tumor angiogenesis [20]. The use of Bev in HHT is off-label but is supported by evidence from several retrospective studies [10, 12, 21–23]. However, many of these studies have focused on epistaxis outcomes [7, 21, 22]. Data regarding the efficacy of Bev in GI bleeding have been limited yet promising. Recent studies have reported that the use of Bev in GI bleeding has led to significantly reduced RBC transfusion requirements, increased mean hemoglobin levels, and reduced GI procedures [9, 12–14]. A multicenter study, the inHIBIT-Bleed study, of 238 patients noted that one year of treatment with Bev resulted in an increase in mean hemoglobin by 3.2 g/dl, decrease in RBC units transfused by 82%, and decrease in iron infusions by 70% [24]. The findings of the present case are consistent with prior studies concerning the use of Bev to manage GI bleeding. Our case adds to the emerging body of literature on the efficacy of Bev for the management of recurrent GI bleeding in patients with HHT.

Although effective, Bev has common and often serious adverse effects (AEs) related to on-target mechanism of action, which may be present in up to 10% of patients [20]. AEs may include arterial thrombosis, hypertension, heart failure, massive GI tract hemorrhage, thromboembolic events, and nephrotic syndrome [20, 23]. Buscarini et al. noted that AEs of Bev affected females more than males with the most common AEs being arthralgias, headache, and proteinuria [25]. Our patient developed hypertension, arthralgias, and lower extremity edema. These AEs were mitigated by dose reduction of Bev to 2.5 mg/kg which is to be administered if the patient's hemoglobin decreased by 1.5 g/dL from its peak. It was determined that the patient would likely require a dose of Bev once every 2 months with serial monitoring of her hemoglobin levels given the severity of her disease.

## 4. Conclusion

In summary, clinicians must consider HHT in the differential for patients with recurrent GI bleeding and AVMs. We recommend the use of Bev in patients with HHT who do not respond to conventional treatment based on its potent efficacy as seen in our case. The AE profile is serious such that dose titration with careful monitoring for vascular complications is critical. Further studies are needed to explore optimal dosing, duration, and long-term safety of Bev.
